# An Introduction to Analytical Challenges, Approaches, and Applications in Mass Spectrometry–Based Secretomics

**DOI:** 10.1016/j.mcpro.2023.100636

**Published:** 2023-08-18

**Authors:** Sascha Knecht, H. Christian Eberl, Norbert Kreisz, Ukamaka Juliet Ugwu, Tatiana Starikova, Bernhard Kuster, Stephanie Wilhelm

**Affiliations:** 1 Omics Sciences, Genomic Sciences, GlaxoSmithKline, Heidelberg, Germany; 2 Chair of Proteomics and Bioanalytics, Technical University of Munich, Freising, Germany

**Keywords:** secretomics, secretome, mass spectrometry, serum-free secretomics, metabolic labeling, cell surface shedding, challenges

## Abstract

The active release of proteins into the extracellular space and the proteolytic cleavage of cell surface proteins are key processes that coordinate and fine-tune a multitude of physiological functions. The entirety of proteins that fulfill these extracellular tasks are referred to as the secretome and are of special interest for the investigation of biomarkers of disease states and physiological processes related to cell-cell communication. LC-MS–based proteomics approaches are a valuable tool for the comprehensive and unbiased characterization of this important subproteome. This review discusses procedures, opportunities, and limitations of mass spectrometry–based secretomics to better understand and navigate the complex analytical landscape for studying protein secretion in biomedical science.

## Highlights

• Overview of challenges, approaches, and opportunities of LC-MS–based secretomics.
• Guidance for unbiased analysis of secreted proteins from cultured cells.
• Strength and weaknesses of different secretomics approaches.

## Graphical Abstract

**Figure:**
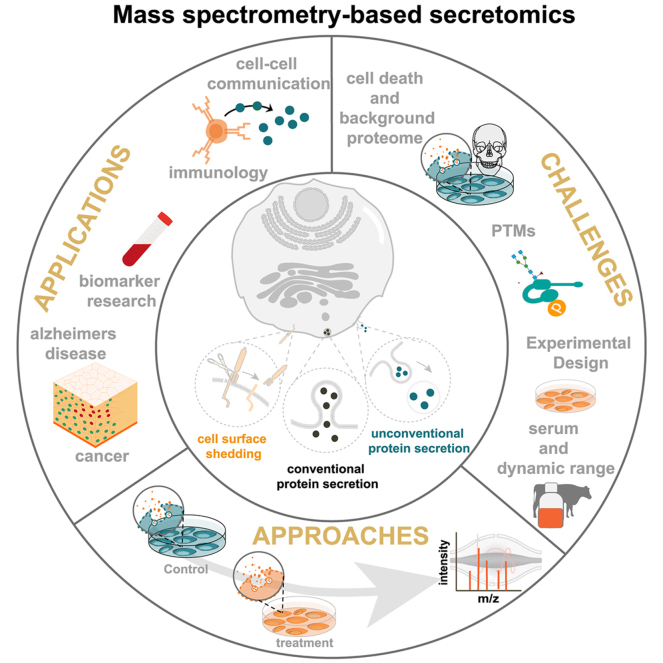

## In Brief

The secretome encompasses a biologically highly relevant and understudied class of proteins that are either actively released by the cell or reach the extracellular milieu *via* tissue leakage. Due to its comprehensive and unbiased nature, LC-MS–based proteomics is an indispensable tool for secretome characterization but requires specific and tailored workflows. This review illustrates the analytical challenges associated with LC-MS–based secretomics, provides a broad survey of established and emerging concepts of secretome analysis, and gives examples of biomedical applications.

Multicellular organisms depend on the dynamic interplay of different organs, tissues, and cell types to sense and respond adequately to changes in the environment. The organization and orchestration of such responses is dependent on an efficient communication of signals between cells. On the molecular level, proteins play a major role as signaling cues for the transmission and reception of signals and can herby act either close by or in far distance.

As such, an important class of proteins are those that are either actively released by a cell into the extracellular environment or reach the extracellular milieu *via* tissue leakage. The entirety of these proteins in the extracellular space are designated as the secretome (1). Functionally, secreted proteins make up a diverse group of proteins covering growth factors, extracellular matrix constituents, cytokines, or hormones.

According to the UniProtKB (accessed December 2022, keyword: secreted), 2097 of 20,401 reviewed proteins in total are annotated as secreted, suggesting that approximately 10% of the human proteome are potentially released *via* the classical secretory pathway or unconventional secretion processes. However, a growing number of experimental data have shown that protein secretion can be uncoupled from the classical endoplasmic reticulum (ER)-Golgi pathway, suggesting that unconventional protein secretion is an important factor which contributes to the active release of proteins into the extracellular space under certain conditions (2, 3, 4, 5, 6, 7, 8, 9, 10, 11). Secreted proteins are in the center of modified signaling pathways of numerous diseases, such as cancer (12, 13), cardiovascular (14), neurodegenerative (15), and chronic liver diseases (16) or obesity (17) and constitute important targets for drugs and diagnostic procedures to track pathogenic processes, disease progression, or pharmacological responses (1).

The systematic investigation of proteins that are actively and passively released by cells is therefore the subject of secretomics. In this review, we provide a broad survey of established and emerging concepts of LC-MS–based secretomics, illustrate the challenges that are associated with the analysis of secreted proteins, and give examples of biomedical applications.

## Challenges of Secretome Analysis

LC-MS–based proteomics has proven to be a key analytical tool for biomedical research and for the investigation of intracellular signaling pathways, protein–protein interactions or for the identification of drug targets, and posttranslational modifications (PTMs). However, a systematic investigation of secreted proteins and intercellular signaling by LC-MS–based proteomics has been less frequently performed because of technical and biological challenges. Nonetheless, LC-MS–based proteomics is a valuable tool for the characterization and in-depth analysis of secretomes, as it gives access to an unbiased and comprehensive view of the entirety of secreted proteins. The possibility to identify thousands of proteins and simultaneously obtain quantitative data differentiates LC-MS–based secretomics approaches from antibody-based readouts that only allow the analysis of a predefined set of proteins and require prior knowledge of the proteins in the sample. A successful investigation of secreted proteins by mass spectrometry, however, requires a deeper understanding of the challenges and limitations which are inherently connected with secretome analysis in general and with the analysis by mass spectrometry in particular. Here, we provide an overview of the main technical and biological challenges that need to be considered for the analysis of secretomes.

### Experimental Design

A major challenge of secretomics experiments is the distinction of truly secreted proteins from the substantial number of background proteins, potentially masking the true biology of the experiment. Data analysis and the interpretation of results therefore benefit from a thoughtful experimental design that should include time-matched untreated control samples. The experimental design should furthermore be based on a well-controlled treatment to call treatment-specific effects and to be able to rule out the impact of cell death. A direct comparison of treated samples with their time-matched controls then allows to evaluate which proteins were differentially released upon a stimulus and which are basally secreted or have leaked into the sample. The experimental design has also a critical influence on the depth and sensitivity of the analysis. For example, hepatocyte model cell lines such as HepaRG cells are highly secretory active upon cytokine treatments (18), allowing the execution of serum-free secretomics experiments in a 12-well plate format and still enable the identification of a multitude of different acute-phase response proteins (ng/ml range). However, the identification and quantification of cytokines, for example, which are usually only present in very low abundances (pg/ml range), may require millions of immune cells to adjust to the detection limit for the LC-MS instrumentation and therefore might require the use of big culture dishes. Protein quantification across different experimental conditions or over different time points to a stimulus or perturbation is another important design element for secretomics experiments. The toolbox of quantitative proteomics approaches includes label-based methods, which are reliant on the labeling with stable isotopes, and label-free strategies, both comprising advantages and disadvantages as reviewed in (19, 20). For protein identification, mass spectrometers are mostly operated in a data-dependent acquisition (DDA) mode in which the most abundant peptides are selected during the MS1 scan for the subsequent fragmentation and MS2 analysis. Depending on the cell type and the biological background of the secretomics experiment, digestion of proteins with low molecular weight and low abundance, such as secreted cytokines, typically result in low peptide numbers. Accordingly, in DDA mode, the low abundance of peptides may result in a low signal intensity in the MS1 scan that prevents the selection for a subsequent fragmentation and MS2 analysis. Data-independent acquisition (DIA) approaches (21, 22) such as SWATH-MS (23) are gaining increasing popularity and have also been successfully applied in secretomics experiments (24). In DIA, all peptides within a defined mass-to-charge (m/z) window are subjected to fragmentation, thus enhancing proteome coverage and reproducibility.

### Cell Death and the Background Proteome

A common observation of secretomics experiments is the presence of hundreds of intracellular proteins, while only a small fraction of proteins are annotated as secreted or extracellular (5, 25). Even under the best culture conditions, the cell culture will never have a 100% viability and a small but not neglectable number of cells will undergo apoptosis or necrosis, which might lead to interferences with the analysis. Sample handling and treatment, for example, excessive washing steps, can further aggravate this problem promoting membrane leakage or cell death. Achieving high cell viability is mandatory for secretomics assays and the contribution of cell death and membrane leakage to the secretome should be monitored. Assessment of the culture quality can be done through quantification of lactate dehydrogenase release into the cell culture supernatant or through trypan-blue staining of cells (25). Furthermore, it has been suggested to use the contribution of highly abundant intracellular proteins to the overall mass spectrometry (MS) signal intensity in the secretome sample, such as structural ribosomal proteins or cytoskeleton components (ACTB, TUBB), as an internal quality measure (2, 26, 27). Increased MS signal intensities of intracellular proteins can be an indication for the contamination of the secretome due to apoptotic processes or compromised cell membranes.

### Serum and the Dynamic Range Problem in MS-Based Secretomics

The majority of secretome studies are performed *in vitro* (28, 29) using mammalian cell culture systems. These cells rely on the addition of (bovine) serum or serum-like supplements to provide a favorable environment for cell growth and normal cellular functions. However, the presence of serum or media supplements poses a major challenge as it leads to a large dynamic range of protein abundances with several orders of magnitude that need to be covered by the mass spectrometer. Highly abundant serum proteins, such as albumin with concentrations of up to 5 g/L in the medium, hamper the detection of cell-derived secreted proteins that typically feature concentrations in a range of low ng/ml (30, 31). To circumvent the dynamic range limitation, the standard approach for secretomics analysis is serum-free cell culture (5, 25, 27, 30, 32). However, a shift from serum-supplemented to serum-free cell culture conditions still bears the risk of residual serum proteins that contaminate the secretome. Even after extensive washing of cells, serum proteins can still be present in the secretome potentially masking very-low abundant proteins. Additionally, it should be considered that residual bovine-derived proteins bear the risk of distorting the results when the raw data are not properly filtered and quantitative information of bovine-derived peptides are included in the downstream analysis. To further improve data reliability, stable isotope labeling with amino acids in cell culture (SILAC) can be applied to label the cellular proteome (33). SILAC is a metabolic labeling strategy that employs stable isotope-labeled amino acids which are added to the cell culture medium and incorporated into proteins by the endogenous protein translational machinery. The application of SILAC labeling (34) can help to discriminate between residual serum contaminants and cell-derived proteins (2, 35) but does not solve the dynamic range problem. However, SILAC labeling requires the use of dialyzed serum to achieve comprehensive labeling of proteins, which was reported to potentially change the cellular proteome (36). Nevertheless, metabolic labeling approaches, like azidohomoalanine (AHA) (37) or labeling of secreted proteins with azido sugars, have been developed that allow for the selective enrichment of secreted proteins under serum-containing cell culture conditions, which thus help to overcome the dynamic range issue. The different secretomics approaches will be discussed later. Another strategy to allow deeper secretome analysis and to overcome the dynamic range issue is the removal of highly abundant serum proteins like albumin by immunoaffinity-based depletion (38) prior to MS analysis. However, depletion of highly abundant proteins can be accompanied by a nonspecific loss of cytokines and other sticky protein species (39), narrowing down the biological relevance of such secretome data. The sample complexity can be further reduced by standard protein fractionation methods using high pH, strong cation exchange, or strong anion exchange fractionation (40, 41). Besides the presence of serum, secretome analysis is further complicated by the complex composition of the basal cell culture medium. The presence of different medium ingredients such as salts, carbohydrates, vitamins, and amino acids can interfere with subsequent sample processing steps and the MS analysis leading to signal suppression. The high dilution and sample complexity often requires high volumes of cell culture supernatant that can be used for subsequent protein concentration and clean-up steps prior to LC-MS analysis. Different strategies have been used to address these challenges: protein precipitation with TCA (30, 42) or SP3 (18), ultrafiltration with low-molecular weight cut-off filters (2) but also lyophilization or speed vacuum centrifugation to concentrate the sample (43) have been applied. However, all these different strategies have strengths and shortcomings. TCA precipitation for example can lead to protein loss, co-precipitation of contaminants like salts or lipids, and can introduce protein modifications. However, TCA precipitation is relatively fast, requires minimal incubation time, it is easily scalable, cost effective, and compatible with a variety of sample types. The use of low-molecular weight cut-off filter can cause protein loss due to selective retention of larger proteins. If proteins of interest have a molecular weight close to the filter's cutoff, they might partially or completely pass the filter, leading to reduced protein yield. This can be particularly problematic for low abundance proteins. In terms of speed, scalability, throughput, and compatibility with chemical-labeling approaches for relative quantification, an SP3-based sample preparation workflow might be beneficial. However, SP3 can lead to loss of low-abundant proteins.

### Posttranslational Modifications

PTMs affect many biological processes and are of importance for the functional diversity of the proteome. Hence it is not surprising that PTMs, such as glycosylations, phosphorylations, sulfations, and citrullinations, also play a crucial role for secreted proteins and changes in PTM decoration are associated with numerous diseases, such as cancer (44), inflammation (45, 46, 47), or congenital (48) and neurological disorders (49). As most PTMs can only be found in the sample in substoichiometric levels, PTM analysis requires an enrichment of the modified proteins or peptides. Whereas glycosylations are well-known PTMs on secreted proteins, the function and biological significance of other PTMs for the extracellular proteome, such as phosphorylations, is understudied and still enigmatic.

By far, protein glycosylation is the most abundant PTM of proteins that is found in all eukaryotic cells and which is involved in a multitude of biological processes such as protein folding, protein solubility, or cell-cell communication (50). As protein glycosylation is a whole field on its own which rapidly evolves, we will only cover this briefly. The attachment of glycan structures with multiple hydroxy groups increases the hydrophilicity of proteins and provides crucial structural and functional properties that are of high significance for diverse biological processes such as blood clotting, cross-linking of cells, or the immune response (51). Alterations in glycosylation profiles are associated with many diseases with diverse clinical representations, such as the congenital disorders of glycosylation or cancer, where glycoproteins are used as biomarkers (52, 53, 54). Hence, the investigation and characterization of the glycan structures in health and disease have become more and more relevant. Even though glycosylations are not a unique feature of secreted proteins and can also be found on intracellular proteins, approximately 66% of all secreted proteins and 87% of type I and type II transmembrane proteins (34) are glycosylated. In contrast, many unconventional secreted proteins (not containing the respective signal peptide) are not glycosylated, which needs to be considered when choosing a suitable secretomics assay, since not all of the presented workflows also allow the exploration of unconventional protein secretion.

LC-MS–based techniques have advanced glycoproteomics research, but challenges remain due to glycan heterogeneity. An overview about common glycoproteomics methods, including different affinity-enrichment techniques and MS workflows to obtain information about the peptide sequence, glycosylation site, and the glycan structure, are reviewed elsewhere (55, 56, 57, 58, 59, 60, 61, 62, 63).

The role of phosphorylations on secreted proteins is still elusive (64, 65). However, owing to the fact that protein phosphorylations are involved in virtually every intracellular signaling process, the appearance of phosphorylations on secreted proteins suggests that these are also associated with specific functions which still need to be discovered. The majority of the extracellular phosphoproteome is generated by a single intracellular kinase, FAM20C (66), which regulates cargo sorting in the TGN through Cab45 (67). However, also extracellular protein phosphorylation has been observed in both health and disease (68, 69, 70). FAM20C kinase substrates are involved in various biological processes including tumor growth, metastasis (71, 72), extracellular protease activity, biomineralization (73), cell motility, proteolytic cleavage, and protease inhibition (74, 75) and were suggested as biomarker candidates for breast cancer (76). A loss-of-function mutation of FAM20C (67) results in the Raine syndrome and is associated with increased ossification resulting in skeletal malformations (71). In contrast, extracellular protein phosphorylations have been observed in the nervous system, where, for example, the phosphorylation of the extracellular protein repulsive guidance molecule b by the secreted tyrosine kinase vertebrate lonesome kinase controls the accurate formation of nervous system circuitry (72). Due to the low abundance of phosphoproteins, specific enrichment steps are required prior to LC-MS analysis. Frequently used and well-established phosphopeptide enrichment techniques are immobilized metal affinity chromatography and metal oxide affinity chromatography (73).

Protein tyrosine sulfations were, so far, only observed on secreted and transmembrane proteins and are catalyzed by Golgi-resident tyrosylprotein sulfotransferases (77). Sulfations increase the polarity and enable electrostatic interactions with basic residues in proteins (78), which plays an important role in protein–protein interactions, cell signaling, adhesion, and immune response (77, 79, 80). They can be enriched using immunoaffinity-based techniques like metal oxide affinity chromatography or strong anion exchange chromatography.

Citrullinations, catalyzed by peptidylarginine deiminases, cause loss of positive charge leading to conformational changes and altered protein–protein interactions (81). Citrullinated proteins, including secreted ones like keratins and fibronectins, play a significant role in immunological processes such as neutrophil extracellular traps formation (81). Analyzing citrullinated proteins *via* MS is challenging due to small mass shifts caused by arginine deimination (74). Enrichment techniques include immunoaffinity-based methods and chemical derivatization followed by solid-phase extraction, liquid-liquid extraction (82, 83), or streptavidin pulldown (75).

#### Cell Surface Shedding

Cell surface shedding of transmembrane proteins, for example, through ADAM proteases, is a major event that contributes to protein secretion in different cell types (68). Additional proteolytic events can be executed through secreted matrix metalloproteinases and cathepsins (69). As such, proteolysis constitutes an important and irreversible PTM that modulates biological processes such as inflammation and innate immunity, adding an additional regulatory level during acute and chronic inflammation by modification of cytokines and chemokines (69, 70). A minor issue that can arise from extensive extracellular proteolysis is the formation of semitryptic peptides which can hamper the identification and quantification of proteins (84) in the secretome. Database searching for all potential nontryptic or semitryptic peptides, for example, with Mascot, Proteome Discoverer, MaxQuant, SEQUEST, or X!Tandem can be time consuming due to an extensive increase of the search space. The integration of computational tools like PROSIT (85) into database search pipelines can help in the identification of nontryptic peptides, thus alleviating this issue.

Another challenge is the identification of soluble protein substrates that are cleaved by secreted proteases, such as MMPs, which is an important posttranslational mechanism to activate or inactivate secreted proteins. Standard secretomics approaches will miss out on such proteolytic events as they do not lead to abundance changes of the protein substrates. This issue can be bypassed through other proteomics approaches, such as chemical enrichment of protein substrates (CHOPS) (86), terminal amine-based isotope labeling of substrates (TAILS) (32), or high-efficiency undecanal-based N termini EnRichment (HUNTER) (87). TAILS enables the identification of proteases and substrates by the analysis of changes in the abundances of protein N termini. Proteolytic cleavage generates protein fragments. One fragment comprises the natural N terminus of the protein and the other fragment comprises the novel protease-generated N terminus. Natural and novel protease-generated N termini as well as ε-amino groups of lysine residues are chemically blocked by dimethylation at the protein level, using light or heavy formaldehyde which allows subsequent mixing of different samples. Mixed samples are then digested with trypsin, hence generating peptides with free N termini, which can be subsequently removed from the sample *via* a reaction with an aldehyde-derivatized polymer. Peptides of protease-generated neo-N-termini remain in the sample as their dimethyl labeling blocks the reaction with the polymer and are analyzed *via* LC-MS/MS. In contrast to TAILS, HUNTER uses a chemical called undecanal to label the free N termini of proteins. Proteins are first purified *via* a SP3 clean-up procedure, followed by protein dimethyl labeling. Excess of dimethyl labeling reagent is then removed with a second SP3 clean-up followed by protein digestion. Resulting peptides are then labeled with undecanal, and undecanal-tagged peptides are removed with a C18 column, while the flowthrough containing peptides of protease-generated neo-N-termini is analyzed by mass spectrometry (87). Even though classic secretomics approaches are limited in their possibility to identify protease substrates, secretomics can aid in the identification of ADAM substrates (77, 78). By taking advantage of the inhibitory effect of the endogenous ADAM protease inhibitor TIMP3, potential ADAM substrates could be identified through comparison of secretomes from TIMP-3 overexpressing cells with secretomes of WT cells. Overexpression of TIMP-3 led to higher TIMP-3 levels in the secretome than the WT cells, while potential ADAM substrates were reduced compared to the WT cells. A similar approach was used to identify BACE1 substrates by overexpression of the protease (79). However, overexpression systems contain the inherent risk of identifying artificial substrates due to overexpression of artifacts like mislocalization.

### Identification of Unconventional Secretion Events

The identification of unconventional secretion events represents a further challenge during secretome analysis. The classical secretion of proteins is based on a short signal peptide (80) that allows for translocation into the ER, the subsequent transport to the Golgi, and the packaging into transport vesicles. Hence, proteins that are secreted *via* the classical secretory pathway can be predicted by computational tools such as SignalP (81). Unconventional secreted proteins lack a signal sequence and bypass the ER-Golgi compartment (82); the signal peptide hypothesis (80) does not hold true in unconventional protein secretion and complicates a prediction. Interestingly, studies have shown that unconventional protein secretion is a non-neglectable contributor to a cell’s secretome with functional implications in the extracellular space (2, 18, 83, 88). For example, HMGB1 was found to be secreted in vesicles, acting as pro-inflammatory factor in the extracellular space (89). Other unconventional secreted proteins like ER-localized chaperones HSP90B1, HSPA5, DNAJC3 have been reported to be transported to the extracellular space during inflammatory conditions (90, 91, 92). However, the verification of an active secretion of such proteins remains challenging as their release can also be a result of cell death and cellular contamination. Different attempts have been made to increase the confidence of such potentially secreted proteins and to exclude contaminants. Most often, a dual approach was applied, in which secretome and proteome data were compared, either in a label-free manner (93, 94) or upon SILAC or iTRAQ labeling (95, 96). The rationale behind this approach is the assumption that actively secreted proteins should have a higher abundance in the secretome than the cellular proteome. Further confidence can be achieved through the comparison of samples with their time-matched controls.

Another important entity that contributes to the unconventional release of proteins are extracellular vesicles (EVs). The generic term EVs describe a heterogeneous group of secreted membrane-enclosed vesicular structures with different sizes, morphologies, and diverse cargo compositions. Based on their intracellular biogenesis, EVs can be broadly categorized into exosomes with a size of 30 to 100 nm and microvesicles with a size of 100 to 1000 nm (97). Initially described as a means of cellular waste disposal (98), EVs are now recognized as an important signal transduction route between cells (99, 100, 101). The isolation and characterization of EVs from cell culture supernatants requires specific purification steps. Frequently applied isolation techniques involve differential ultracentrifugation, density gradient centrifugation, precipitation with PEG, immunoaffinity-based techniques, and size-exclusion chromatography (102). Even though different EV enrichment techniques exist and have been successfully applied, the analysis of EVs is not trivial. Typically, high cell numbers are required and the EV yields strongly depend on the cell type and the applied isolation techniques. Moreover, EV analysis demands serum starvation or use of EV-depleted serum. The growing interest in EVs and the shortcoming in retrieving information about their respective cargos lead to the development of online repositories, which collect experimental data from EV studies and allow to query EV-based cargo. ExoCarta is a manually curated compendium of exosomal cargos, comprising 41,860 protein entries from different species (103). Whereas ExoCarta is limited to exosomal cargo, Vesiclepedia covers cargo from all classes of EVs (104), comprising 349,988 protein entries from 41 different species.

## Analytical Approaches for Probing the Secretome

### Serum-Free Secretomics

**Fig. 1:**
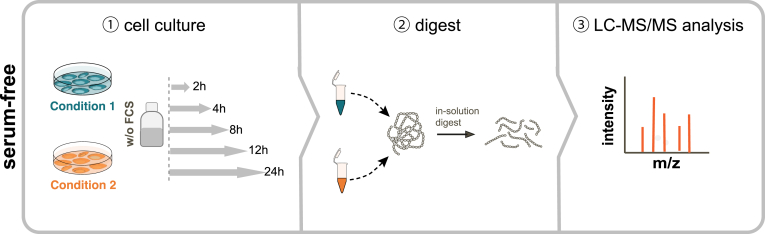
**Schematic representation of the serum-free secretomics workflow.** ① Protein secretion is probed by collecting cell culture supernatants under serum-free conditions for a defined treatment time range and is subsequently cleared to remove potential cell debris by centrifugation or by using a 0.45 μm syringe filter. ② Proteins are digested, and the resulting peptide samples are ③ analyzed by LC-MS/MS.

As discussed in the previous section, the majority of secretome studies are performed *in vitro* using mammalian cell cultures that are usually grown in the presence of up to 20% fetal calf serum, which poses an analytical challenge for LC-MS–based read-outs. The most widespread approach for the analysis of secretomes *via* LC-MS–based proteomics is the direct quantification of proteins from serum-free cell culture supernatants (5, 18, 25, 27, 30, 32) (Fig. 1 and Table 1). Cells are cultured in the presence of serum, and protein secretion is probed under serum-free conditions by shifting the serum-containing culture medium to the basal medium without fetal calf serum. For the removal of residual serum proteins, it is advisable to wash the cells two to three times carefully with the prewarmed basal medium prior to incubation. Label-free secretomics experiments are carried out using phenol-red–free medium, to further minimize analytical interference during the LC-MS/MS analysis as phenol-red, when not removed through appropriate sample preparation methods, can lead to a charged peak in the chromatogram. The conditioned medium containing the released proteins is then collected and either centrifuged or filtered to remove detached cells and cellular debris. The main determinant of the incubation time in serum-free medium is to find a balance between achieving good proteomic coverage and reducing the negative impact of nutrient and growth factor deficiency on protein expression. Label-free secretomics experiments are easily scalable from dishes to multiwell plates, depending on the cell type, cell number, stimulus, and the secretory activity of the cells and are thus suitable for experiments using, for example, primary or FACS sorted cells (105). However, of utmost importance for the experimental design is the implementation of suitable controls, for example, unstimulated or untreated cells, which help to differentiate basal secretion events from stimulus-induced secretion. Additionally, secretome samples from untreated cells help to judge the cell health and viability and as such, the secretome quality by comparison of the quantitative lactate dehydrogenase values between the controls and the treated samples. The proteins are then subjected to a bottom-up proteomics sample preparation workflow (Fig. 1). The downstream processing of the secretome samples can be done through different approaches, such as protein precipitation or ultrafiltration. However, the selection of sample preparation methods relies on the experimental setup and the sample volume. In general, precipitation-based methods such as classical TCA precipitation or carrier-aided TCA precipitation were successfully applied, for example, for macrophages and dendritic cells for the investigation of secretion upon activation with lipopolysaccharides (LPS) (30). But also filter-aided sample preparation, SP3, and urea-based methods have been robustly used for secretome studies of murine islets of Langerhans, liver cell lines, and LPS-stimulated macrophages (5, 18, 41, 106, 107).

**Table 1:** Mass spectrometry–based approaches for probing the secretome

Secretome approach	Turn-around time	Required number of cells	Compatible with FCS	Special reagents	Strengths and disadvantages	References
Serum-free	∼2 days	∼10^5^	No	Phenol-red–free basal medium	- ease of implementation, no requirement of elaborate and sophisticated enrichment techniques - allows testing of multiple biological conditions in a short period of time - easily scalable from dishes to multiwell cell culture plates - versatile, compatible with different cell numbers, sample preparation methods (TCA precipitation, FASP, SP3), and SILAC labeling - Facilitates the analysis of cell surface shedding and unconventional secretion events - requires shift to serum-free culture conditions - only partly suitable for tracking of transcriptional dependent processes - serum-free conditions may affect cell viability and responsiveness - activation of cells with proteins or ligands that require serum can be difficult - only partly suitable for primary cells - requires optimization of medium supplements	(4, 5, 18, 25, 105, 106, 131, 132, 133)
AHA labeling	∼3–4 days	∼ 10^6^	Yes	Azidohomoalanine	- Compatible with serum, thus also suitable for primary cells - Facilitates the analysis of cell surface shedding	(37, 115, 134, 136, 138)
				SILAC amino acids	- Allows stringent washing steps to remove serum contaminants - Labeling allows distinction between cell derived and serum proteins	
				Nonstandard medium formulation (no methionine)	- allows the analysis of cell surface shedding and unconventional secretion events	
					- compatible with SILAC labeling	
				Alkyne-agarose beads	- Potentially cytotoxic, demands a precise optimization of cell culture conditions and pulsing times	
					- methionine depletion step can disturb cells	
					- Only newly synthesized proteins can be analyzed, not suitable to analyze the secretion of proteins which are stored in secretory granules	
					- Limited in throughput due to more extensive sample work up compared to serum-free approaches	
					- copper-CLICK-chemistry-based pulldown requires long reaction times of approximately 18 h	
					- Requires high cell numbers and nonstandard media formulations without methionine	
					- vulnerable to perturbations of the cellular translation machinery	
					- click reaction can be influenced by the complexity of the sample matrix (plasma protein binding of reagents)	
**Azidosugar labeling (SPECS/hiSPECS)**	∼4 days	∼ 10^6^	Yes	N-azidoacetyl mannosamine-tetraacylated	- Compatible with serum, thus also suitable for primary cells - Facilitates the analysis of cell surface shedding - Allows stringent washing steps to remove serum contaminants - compatible with SILAC labeling	(24, 137, 139)
				DBCO-alkyne beads	- Limited in throughput due to extensive sample workup	
					- Requires 48 h treatment with ManNAz to label proteins	
					- Requires high cell numbers	
					- Only glycosylated proteins can be analyzed	
					- Dependent on a functional protein-glycosylation machinery	
					- unconventional secretion events of nonglycosylated proteins are missed	
Proximity labeling	>> 7 days, including cell line generation	∼10^5^	Yes	Plasmids or lentiviral constructs	- Compatible with serum - Compatible *in vivo* and with primary cells - Allows investigation of organ and cell type–specific protein secretion in live animals	(122, 124, 125)
				Biotin	- High temporal resolution due to fast labeling kinetics	
					- Requires cloning and transfection of cells or adenoviral delivery	
					- Labor intensive and time consuming	
					-Overexpression can lead to artificial high protein abundances and mislocalizations	

FCS, fetal calf serum.

**Fig. 2:**
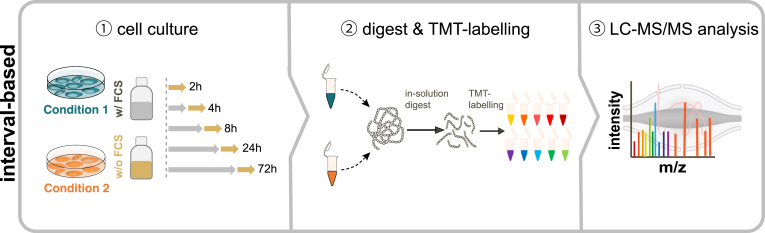
**Schematic representation of the interval-based secretomics workflow.** ① Treatments are performed in the presence of serum and secretion is probed in 2 hour serum-free collection windows. Serum-free supernatants are cleared to remove potential cell debris by centrifugation through a 0.45 μm filter plate. ② Proteins are digested; peptides are chemically labeled with isobaric TMT reagents and are subsequently pooled. ③ Pooled peptide samples are analyzed by LC-MS/MS.

The omission of serum improves the dynamic range and facilitates the identification of low abundant secreted proteins that would be otherwise masked by high abundant serum proteins. However, the analytical improvements that come along with serum deprivation are at the same time a burden for the cells: serum-free cell culture conditions can lead to alterations in protein secretion, phosphorylation, and expression levels of proteins, or in general compromise cell viability and result in a stressed cell state (2, 37, 108, 109, 110). Another major drawback is the time limitation in which the secretion can be probed, usually covering timeframes of only a few hours. Prolonged incubation times in serum-free medium can compromise cellular integrity, narrowing down the biological relevance of such secretome data (2, 5, 111). The analysis of cellular responses that are exceeding those time limitations, for example, transcriptional dependent processes, is therefore limited. To address these limitations, an interval-based secretomics approach has been proposed as an elegant alternative for current serum-free secretomics methods (18). The interval-based secretomics approach allows secretomics analysis from cells treated in the presence of serum by probing the protein secretion rate into the supernatant in short intervals of serum-free medium (Fig. 2). The limited time window in which cells are maintained in serum-free medium extends the experimentally covered time range to multiple days without a negative impact on cell viability. Moreover, the interval-based secretomics approach employs a tandem mass tag (TMT)-based isobaric labeling strategy, allowing the combination of multiple time points, treatments, or treatment combinations into one TMT experiment, thus ensuring complete quantitative data for all identified proteins at all conditions.

### Secretome Analysis Utilizing Metabolic Labeling Approaches

**Fig. 3:**
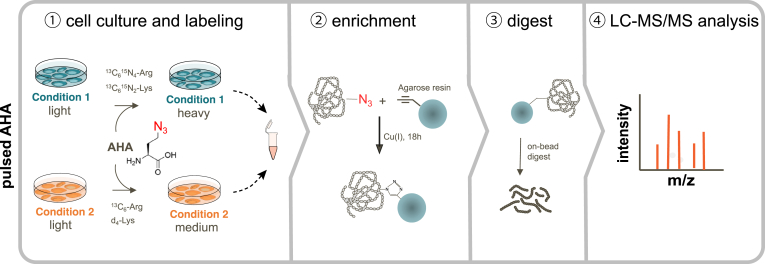
**Schematic representation of the enrichment of secreted proteins using a dual labeling approach with azidohomoalanine in combination with stable isotope labeling by amino acids in cell culture.** ① To analyze proteins in serum-containing media, a pulse labeling with AHA in combination with SILAC is performed. ② Newly synthesized proteins carrying the AHA label and that are secreted into the cell culture supernatant are enriched through covalent coupling to an alkyne-activated agarose resin *via* a Cu(I)-catalyzed azide-alkyne cycloaddition (CuAAC-mediated click chemistry). The covalent coupling to the agarose resin allows stringent washing for the removal of high abundant serum proteins and other contaminants. ③ Resin-bound proteins are digested and the resulting peptide samples are ④ analyzed by LC-MS/MS. AHA, azidohomoalanine; SILAC, stable isotope labeling with amino acids in cell culture.

**Fig. 4:**
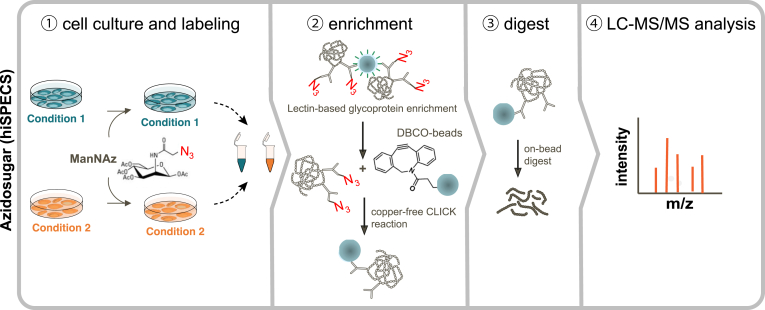
**Schematic representation of the enrichment of secreted proteins by metabolic-labeling with azido sugars using the hiSPECS approach.** ① Protein secretion is probed by collecting cell culture supernatants from ManNAz-labeled cells. ② Glycoproteins are first enriched from the serum-containing cell culture medium *via* a lectin-based glycoprotein enrichment using concanavalin A beads. Beads are washed and the glycoproteins are eluted through competition with methyl-alpha-D-mannopyranoside. The azido-glycoproteins are then selectively captured from the bulk of serum-derived glycoproteins *via* covalent binding to magnetic dibenzylcyclooctyne (DBCO)–alkyne beads through copper-free click chemistry, which enables a stringent wash to remove unspecific bound serum proteins. ③ Bead-bound proteins are digested and the resulting peptide samples are ④ analyzed by LC-MS/MS. hiSPECS, high-performance secretome enrichment with click sugars.

To mitigate the shortcomings of serum-free cell culture, metabolic labeling approaches were developed that are compatible with the use of serum and do only minimally interfere with cellular processes. Metabolic labeling exploits the cellular enzyme machinery to label proteins with affinity tags that can then be used to selectively separate those proteins from the high abundant serum proteins in the cell culture medium. Basically, two protein labeling strategies are commonly used for the selective enrichment of secreted proteins in serum-containing media, pulsed azidohomoalanine (pAHA) labeling (Fig. 3), and labeling with azido sugars (Fig. 4).

#### Metabolic Labeling with AHA

Eichelbaum *et al*. used a dual labeling approach with AHA, an analog of methionine that carries an azide group (37) in combination with SILAC (112) (Fig. 3 and Table 1). The unnatural amino acid AHA is used by the endogenous methionine tRNA and incorporated into the protein backbone of newly synthesized proteins instead of methionine (113). Newly synthesized proteins that are secreted into the cell culture supernatant and that are carrying the AHA label can be distinguished from the unlabeled (serum) proteins and are enriched through covalent coupling to an alkyne-activated agarose resin *via* a Cu(I)-catalyzed azide-alkyne cycloaddition (CuAAC-mediated click chemistry). The covalent coupling to the agarose resin allows stringent washing for the removal of high abundant serum proteins and other contaminants. In order to enable successful introduction of AHA, a methionine depletion step before the addition of the AHA-containing medium (which contains AHA in high excess) is required and thus nonstandard media formulations without methionine will be needed. Metabolic labeling of secreted proteins and the subsequent enrichment from the cell culture supernatant requires more extensive sample workups which can introduce technical variability, for example, due to sample loss during wash steps or different efficacies during the click reactions. Moreover, enriched samples are measured separately in multiple runs, which reduce the throughput, limits the measurement precision, potentially leads to data incompleteness, and lowers the quantification accuracy. To mitigate these shortcomings and to reduce the experimental error, improve the quantification accuracy, and data completeness, AHA labeling is often combined with SILAC. Moreover, SILAC labeling enhances the data reliability as it further helps to discriminate cell-derived proteins from the contaminating serum proteins. However, the introduction of SILAC requires at least five cell doublings to achieve a high incorporation state in cells (33) and thus cannot be applied to nondividing primary cells (24). While SILAC labeling is normally well tolerated, AHA labeling can induce cellular toxicity and thus demands a precise optimization of cell culture conditions and pulsing times (114).

A recent study by Vargas-Diaz *et al*. addressed the long reaction time and extensive sample workups required for copper-CLICK-chemistry–based pulldown of AHA-labeled proteins (115). They transferred the enrichment procedure to an automated AssayMap Bravo Platform, improving speed and reproducibility with a turnover time of 3 h. The copper-click reaction was replaced with a copper-free alkyne-azide click reaction using a dibenzylcyclooctyne (DBCO) moiety ([3+2] cycloaddition of the AHA-azide moiety with a strained cycloalkyne (116)), significantly reducing the reaction time from 18 h to 1 h. To simplify and automate the enrichment process, they utilized a molecule connecting the DBCO group with a Dde group and biotin group *via* a PEG-4 chain.

#### Metabolic Labeling with Azido Sugars

Azidosugar-labeling takes advantage of the fact that approximately 66% of all secreted proteins and 87% of type I and type II transmembrane proteins are glycosylated (34). Kuhn *et al*. made use of this observation and developed the secretome protein enrichment with click sugars (SPECS) and successfully demonstrated its compatibility with primary neurons (34). SPECS allows the selective enrichment of secreted glycoproteins from the bulk of serum proteins. Cells are grown in a culture medium in the presence of tetraacetyl-*N*-azidoacetyl-mannosamine (ManNAz), which passively diffuses through the cell membrane. In the cytosol, cellular esterases cleave off the acetyl groups and the sugar is metabolically converted to *N*-azidoacetyl-sialic acid, which is then incorporated into terminal positions in *N*- and *O*-linked glycans. In the next step, cell-derived glycoproteins in the secretome are biotinylated *via* a click reaction with cycloalkyne dibenzylcyclooctyne (DBCO-PEG12-biotin) and are subsequently enriched *via* a streptavidin pulldown (116, 117).

A disadvantage of the initial SPECS method is the requirement of up to 40 × 10^6^ cells per sample, which is not always feasible, especially when primary cells are used. Additionally, the turnaround time of the SPECS workflow is approximately 6 days, consisting of several ultrafiltration steps to remove the excess of free ManNAz and DBCO-PEG12-biotin and extensive sample fractionation. Hence, the workflow is quite labor intensive and the ultrafiltration leads to a loss of proteins. To tackle these shortcomings, Tüshaus *et al*. set out to improve some major steps of the SPECS method to increase the feasibility for low sample amounts (24). The result was the high-performance secretome enrichment with click sugars (hiSPECS), a miniaturized version of the SPECS method that offers higher sample throughput and reduces the required input sample amount, from 40 × 10^6^ cells per sample to 1 × 10^6^ cells per sample (24) (Fig. 4 and Table 1). After metabolic labeling of proteins with ManNAz, the glycoproteins are first enriched from the cell culture medium with concanavalin A beads. The beads are washed and the glycoproteins are eluted through competition with methyl-alpha-D-mannopyranoside. Then, the azido glycoproteins are selectively captured from the bulk of serum-derived glycoproteins *via* covalent binding to magnetic DBCO–alkyne beads through copper-free click chemistry, which enables a stringent wash to remove unspecific-bound serum proteins. After on-bead digestion, peptides are either analyzed *via* DDA or *via* DIA. This enabled the characterization of secretomes of primary brain cell types from single mice.

#### General Constraints of Metabolic Labeling Strategies

Although elegant, metabolic labeling strategies have inherent weaknesses. In general, these methodologies rely on specific media compositions and reagents that could potentially lead to alterations in cell growth and viability (118) or are not compatible with the chosen cell type. Moreover, they exhibit biological limitations: Azidosugar-based enrichment methods miss unconventional secretion events of nonglycosylated proteins. pAHA labeling approaches are vulnerable to perturbations of the cellular translation machinery and proteins which were synthesized before the AHA pulse, for example, pre-existing secretory granules, cannot be specifically enriched and will be missed. Hence, AHA labeling is biased towards proteins with high turnover rates. Additionally, AHA labeling in combination with the necessary methionine starvation can potentially disturb cellular signaling pathways. AHA can introduce changes in protein expression since its incorporation into proteins is slower than methionine (119). In a recent paper, the effects of AHA labeling on gene and protein expression were elucidated, revealing profound changes on both transcriptome and proteome level together with AHA-induced cellular stress, due to protein misfolding and reduced translation rates (120). Interestingly, they also found that more than 30% of the proteins identified in the secretome were upregulated or downregulated, which demonstrates that AHA labeling has significant quantitative and qualitative impact on the secretome composition and leads to unpredictable bias in the data. Technical limitations lie in the click-chemistry–driven pulldown of labeled proteins which can be influenced by the complexity of the sample matrix. Biotinylation reagents that contain long lipophilic PEG linker between the biotin group and, for example, the DBCO-head group can potentially bind to plasma proteins and negatively affect the overall biotinylation reaction (121).

In summary, metabolic labeling allows the enrichment and relative quantification of secreted proteins in the presence of serum and is hence suitable for cell lines that are susceptible to serum-free culture conditions, such as primary cells. The presence of serum circumvents the negative effects of serum starvation and improves culture quality. However, metabolic labeling does not solve or eliminate the contribution of cell death to the secretome, since the enrichment step will not differentiate between truly secreted proteins and contaminating intracellular proteins.

### Characterization of Tissue-Specific Protein Secretion in Live Animals Using Proximity Labeling Approaches

**Fig. 5:**
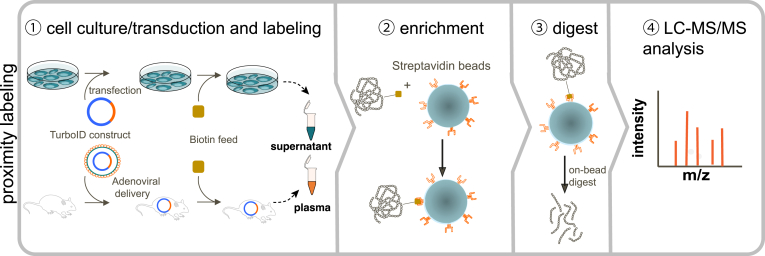
**Schematic representation of the enrichment of secreted proteins by proximity labeling in cell culture or animals.** ① Biotin ligases (BioID, TurboID, miniTurbo) are targeted to the secretory pathway, for example, by tagging the translocon SEC61 in the endoplasmic reticulum (ER) membrane or by targeting to the ER lumen using a transfection or viral transduction strategy. In the ER, secretory proteins are biotinylated prior to their secretion into the extracellular space (cell culture medium or plasma). ② Biotinylated proteins can then be enriched using streptavidin affinity purification. ③ Bead-bound proteins are digested and the resulting peptide samples are ④ analyzed by LC-MS/MS.

In addition to the survey of secretomes *in vitro*, there is a growing body of interest to characterize protein secretion of distinct organs and tissues *in vivo*. However, current secretomics approaches are limited to *in vitro* and *ex vivo* models which are sometimes not representative for real *in vivo* biology (122). The development of proximity-labeling techniques in 2012 (123) enabled the study of tissue-specific protein secretion in animals, which has been successfully applied by several independent research groups (122, 124, 125, 126). Proximity labeling makes use of engineered enzymes that are genetically fused to a protein of interest. The proximity labeling enzyme then generates intracellular short-lived reactive molecules, like radicals or esters, from inert small-molecule substrates that provoke an *in situ* labeling of proximal proteins. Different proximity labeling enzymes have been engineered to enable the *in situ* tagging of proteins, such as ascorbate peroxidase 2 (APEX2), horseradish peroxidase, or biotin ligases (BioID, TurboID, miniTurbo) (123, 127, 128, 129). Biotinylation is a two-step reaction, where the biotin ligase generates the reactive biotin intermediate biotinoyl–adenosine monophosphate (biotin–5ʹ-AMP) which reacts with lysine side chains on proximal proteins (123, 130). Biotinylated proteins can then be enriched using streptavidin affinity purification and subsequently identified through mass spectrometry. Originally, proximity labeling was developed as complementary assay to classical affinity-purification techniques allowing the characterization of protein–protein interactions. However, to bypass the limitation of current secretomics approaches that are confined to *in vitro* or *ex vivo* models, proximity labeling has also been established to characterize protein secretion *in vivo* (122, 124, 125) (Fig. 5 and Table 1). This was achieved by targeting these biotin ligases to secretory pathways, for example, by tagging the translocon SEC61 in the ER membrane or by targeting BioID to the ER lumen, where a biotinylation of secretory proteins can be achieved prior to their secretion into the extracellular space. Proteins that are tagged with biotin can then be easily tracked and enriched from body fluids like blood plasma.

## Applications of Secretomics Strategies in Biomedical Research

The above described secretomics strategies have enabled a systematic investigation of secreted proteins and have contributed to a better understanding of the complexity and the dynamics of the secretome as well as the physiological function of secreted proteins in diverse biological settings. This section gives an overview of key publications and the latest advances and discoveries of quantitative secretome analysis applied in different biomedical areas. We have collated studies where secretomics was used to answer immunology-driven questions and to explore general messaging principles across different cell types, helped to identify novel biomarkers in cancer and Alzheimer’s research, or enabled the characterization of protein secretion events modulated in disease.

### Applications of Serum-Free Secretomics Approaches

Serum-free secretomics approaches have facilitated systems-level investigations of intercellular signaling structures and have improved our understanding of how cells exchange information in different biological contexts.

In 2013, Meissner *et al*. presented a quantitative MS-based proteomics workflow that enabled a direct identification of secreted proteins in serum-free cell culture supernatants from LPS-activated bone marrow–derived mouse macrophages (5). Through the comparison of the secreted protein repertoire of LPS-treated WT primary macrophages with the secretomes of MyD88-, TRIF-, or MYD88-TRIF-double KO cells, this study enabled a systematic dissection of the TLR4 signaling pathway. Moreover, the study underpinned the strength of serum-free secretomics approaches to be suited for the detection of unexpected extracellular proteins, showing that many proteins were actually released by unconventional protein secretion pathways, including cell surface shedding events. Furthermore, the study compared the secretome data with transcriptome data and observed an uncoupled response for different gene populations in their transcriptomic and secretomic progression.

A more system-wide perspective of how immune cells communicate to protect against pathogens was further established by Rieckmann *et al*. in 2017 (105). Through a combination of flow cytometry with serum-free secretome and total proteome measurements, 28 human hematopoetic cell populations derived from human donors were characterized in steady state and during activation. The study determined general messaging principles and found that antigen-presenting cells are at the top of an intercellular signaling hierarchy, as they increase their capacity to send information in response to changing conditions. In contrast, cells with cytotoxic functions decreased their hierarchy upon changing conditions. Moreover, the study gained insights into general messaging principles, for example, that specificity in intercellular signal transduction is achieved through restriction of communication to a limited number of sending or receiving cell types for any given cytokine.

Another immunological application of serum-free secretomics helped in the understanding of tumor necrosis factor (TNF)-induced cell death modes such as apoptosis and necroptosis (131). Chronic inflammatory TNF-induced cell death contributes to a range of inflammatory diseases like psoriasis or rheumatoid arthritis. A time-resolved serum-free secretome analysis of lymphoma cell lines and primary human macrophages upon induction of TNF-mediated apoptosis and necroptosis determined similarities and differences between apoptotic and necroptotic cell death. While cell surface shedding occurred in both modes of cell death, necroptotic cells reduced their conventional secretion of cytokines but increased the release of lysosomal proteins. In contrast to necroptosis, apoptotic cells were marked through the release of histones.

Secretomics has proven valuable in various areas, including understanding cell communication and disease modulation. Deshmukh *et al*. employed a serum-free secretomics approach to investigate the skeletal muscle secretome in C2C12 myotubes (25). They identified novel myokines and observed distinct secretion patterns between normal and insulin-resistant myotubes. Insulin resistance led to the downregulation of IGFBP7 and increased secretion of cytokines, along with reduced levels of BMP1, a regulator of muscle growth activation. In the study of human brown fat cells, secretome analysis revealed differences in the secreted protein repertoire between brown and white adipocytes (132). These proteins included hormones, growth factors, extracellular matrix proteins, and complements, indicating distinct metabolic functions for each cell type. Notably, EPDR1 was found to be differentially secreted in brown fat cells. Further experiments using siRNA knockdown of EPDR1 demonstrated its role in the metabolic response to adrenergic signaling. Whole-body knockout of Epdr1 in mice confirmed its importance in the development of functional thermogenic adipocytes and its endocrine impact on whole body metabolism.

While most of the serum-free secretome studies were based on a label-free protein quantification approach to assess the differences between two or more biological states, only a few studies so far have implemented isotope tags for a relative quantification of proteins, like, for example, iTRAQ or the more popular TMT. A TMT-based labeling strategy was used by Wang *et al*. who performed serum-free secretome analysis of 13 neuroendocrine (NE) tumor cells and dissected the communication pathways that drive oncogenesis (133). The group studied cancer secretomes of small cell lung cancer to understand pulmonary NE tumor biology. They analyzed secretomes from tumor cells of ASCL1high and NEUROD1high subtypes and found that lineage-specific transcription factors drive distinct secretomes. The analysis identified IGFB5 as a direct transcriptional target of ASCL1, making it a potential secreted marker for ASCL1high small cell lung cancers. This study highlights the usefulness of secretomics in exploring potential cancer biomarkers.

### Applications of Metabolic Labeling Approaches

As an alternative to serum-free secretomics approaches, bioorthogonal metabolic-labeling approaches have been successfully exploited for the in-depth analysis of secretomes from various cell lines and primary cells (37, 115, 134, 135, 136). Here, we show the latest applications of these metabolic-labeling approaches for the analysis of secretomes.

#### Applications of pAHA for the Analysis of Secreted Proteins

The pioneering application of metabolic labeling with AHA for the selective enrichment of secreted proteins from cell culture supernatant was introduced by Eichelbaum *et al*. in 2012 (37). By comparing enriched and nonenriched samples, they demonstrated the superiority of the enrichment step, as it revealed a much larger number of quantified proteins (684 *versus* 22 human proteins). They further utilized this approach to study the secretomes of different cell lines and observed rapid changes in protein secretion upon serum withdrawal. The method was also employed to investigate protein secretion kinetics in LPS-stimulated macrophages, revealing both known and previously unreported secreted proteins involved in various biological processes. This study showcased the versatility and potential of the metabolic-labeling approach in studying protein secretion dynamics and identifying new biomarkers.

In another study, the same group used proteome, transcriptome, and secretome data, to unravel the temporal dynamics of LPS-stimulated RAW 264.7 cells (135). They found that within a 3-h time course, macrophages undergo rapid proteomic changes that cannot be captured by full or pulsed SILAC (pSILAC) labeling alone. In contrast, pAHA labeling allowed detection of newly synthesized and secreted proteins and revealed the correlation between protein synthesis, secretion, and transcriptional response. Notably, the study identified not only canonical pro-inflammatory proteins but also immune-modulating proteins induced by LPS and TLR4 downstream signaling. Some proteins showed increased fold changes in the secretome without corresponding changes in transcript or intracellular protein levels, suggesting enhanced secretion through unconventional pathways. The pAHA and pSILAC secretomics approach also revealed cell surface shedding events, highlighting the complexity of immunological responses.

The pAHA and pSILAC labeling approach was also used to compare secreted proteins from white and brown murine adipocytes derived from the stroma-vascular fraction (134). AHA labeling enabled the comprehensive characterization of secreted proteins in steady state and after stimulation with norepinephrine (NA), which triggers physiological responses in adipose tissue. White adipocytes primarily secreted proteins involved in carbohydrate metabolism, while no specific protein class dominated the secretion from brown adipocytes. NA stimulation altered the secretory output of both cell types, with white adipocytes shifting towards adipogenesis and oxidative resistance proteins and brown adipocytes showing increased secretion of known adipokines and the discovery of novel batokine candidates. Additionally, the analysis revealed unexpected enrichment of tricarboxylic acid proteins in NA-treated white adipocyte secretomes.

#### Applications of Metabolic Glycan Labeling with Azido Sugars for the Analysis of Secreted Proteins

The second method that exploits metabolic labeling of proteins and combines it with a selective CLICK-chemistry–driven pulldown, called SPECS, was established by Kuhn *et al* in 2012 (34). Kuhn *et al*. used this approach for the characterization of brain cell secretomes with a special focus on the determination of cell surface shedding events by the protease BACE1, a key drug target for Alzheimer`s disease.

Witzke *et al*. used the SPECS approach for the unbiased secretome analysis of activated T-cells (137). The authors found known and novel proteins to be secreted upon activation of Jurkat cells with ionomycin and phorbol-12-myristate-13-acetate; however, classical T-cell cytokines like IL2 or IFN-y were not identified, most likely due to their extreme low concentration.

A major bottleneck of the SPECS method is the requirement of high cell numbers, which is especially challenging for the analysis of primary cells with limited access. The original SPECS methodology was further improved and miniaturized by Tüshaus *et al*. by developing the hiSPECS method (24). hiSPECS enabled the identification of new BACE1 substrates in the four different cell types and provided new insights into the biology of brain cells. Protein shedding quantitatively differed between the different brain cell types and provided an additional layer of control. Of note, the secretome data suggested that cell type–specific protein secretion contributed to functional differences between the four cell types and that these differences in protein secretion are not fully attributable to cell type–specific expression patterns as the cell type–specific proteins were equally expressed in all cell types. Moreover, the authors applied hiSPECS to cortico-hippocampal brain slices that preserve the complexity of diverse brain cell types and thus demonstrate that secretome analysis can even be done with *ex vivo* brain slices.

### Application of Proximity Labeling to Track Protein Secretion *In Vivo*

The introduction of proximity-labeling techniques enabled the first *in vivo*-secretome studies with tissue-specific resolution. Kim *et al*. developed the *in situ* secretory protein labeling *via* ER-anchored TurboID and applied this approach to identify biotinylated secreted proteins in mouse blood plasma. *In situ* secretory protein labeling *via* ER-anchored TurboID makes use of the expression of a fusion protein which is composed of the ER protein Sec61 and the biotin ligase TurboID (Sec61b-TurboID). They validated this approach through labeling of liver secretory proteins and tracked liver secretome–specific changes associated with induced systemic insulin resistance. A similar analytical strategy was used by Liu *et al*. to deconvolute the *in vivo* secretome of endothelial cells and skeletal muscle in a mouse model (the “secretome mouse”) expressing an ER-targeted TurboID biotin ligase (124). Wei *et al*. generated a proteomic atlas of hepatocytes, myocytes, pericytes, and myeloid cell secretomes by the enrichment of biotinylated-secreted proteins from plasma samples of mice (125). Moreover, they characterized liver-specific alterations of the secretome in mice that are associated with a high-fructose, high-sucrose diet. To study interorganellar communication structures in *Drosophila* and mice, Droujinine *et al*. expressed the biotin ligase BirA∗G3 and identified 51 muscle proteins in the *Drosophila* head and over 200 fat body–secreted proteins targeted to the legs and muscles, suggesting distal functions of these proteins as part of an extensive interorgan communication network (126). Moreover they translated the BirA∗ approach to mice by using an ER-targeted BirA∗G3 construct expressed in kidney teratomas to identify known hormones and signaling proteins.

## Concluding Remarks and Outlook

The characterization of secreted proteins using MS-based proteomics approaches represents a challenging field. In this review, the recent advances and successes in the field of MS-based secretomics along with the challenges associated with the analysis of secreted proteins were compiled. Different approaches that can be applied for secretome analysis were outlined and the different constraints that are inherently connected with each of the different secretomics approaches were discussed. In addition, we have highlighted key publications and the latest advances and discoveries in quantitative secretome analysis applied in different biological settings, such as immunology and biomarker research.

When secretome analysis is to be considered, the first decision that must be made is the choice of the secretomics strategy that should be applied, considering the strengths and shortcomings of each of the presented approaches. We believe that a serum-free secretomics strategy is the first approach to be tested, since it is the most versatile and provides the highest sample throughput with the least extensive sample workup compared to metabolic-labeling approaches. Moreover, with the introduction of the interval-based serum-free secretomics approach (18), some shortcomings of serum-free workflows have been addressed, which now enables the monitoring of transcriptionally regulated secretion processes and renders serum-free workflows even more attractive. In addition, serum-free secretomics approaches are suitable for covering a broader range of different secretion events that would otherwise be missed with metabolic-labeling approaches, including the unconventional secretion of nonglycosylated proteins, proteins secreted *via* exosomes, or the secretion of proteins that are stored in secretory granules. Furthermore, serum-free approaches enable the study of secondary compound effects, for example, as a result of compound metabolization. This can even include small molecule treatments that would normally interfere with the metabolic labeling of proteins (*e.g.* inhibition of protein glycosylation), hence preventing the use of metabolic labeling approaches.

In conclusion, we believe that the analysis of secretomes will become a standard approach and an essential tool to understand the secretory environment, paracrine and autocrine messaging processes in health and disease, and will provide new potential therapeutic purposes and diagnostic perspectives. Secretomics will benefit from future advancements in MS-based technology that will increase sensitivity and throughput and will allow the analysis of secreted proteins from fewer numbers of cells. In summary, the development of new and the improvement of the current secretomics approaches will open up the field for new basic research and biomedical applications and have the potential to markedly support and accelerate modern drug and biomarker research.

## Conflict of interest

S. K. and H. C. E. are employees of GSK. H. C. E. is a shareholder of GSK. B. K. is a cofounder and shareholder of OmicScouts and MSAID. He has no operational role in either company. Neither company funded the presented work. The funders had no role in the design of the study; in the collection, analyses, or interpretation of data; in the writing of the manuscript; or in the decision to publish the results. The authors declare that they have no conflicts of interest with the contents of this article.

## References

[1] Uhlen M., Karlsson M.J., Hober A., Svensson A.S., Scheffel J., Kotol D. (2019). The human secretome. Sci. Signal..

[2] Villarreal L., Mendez O., Salvans C., Gregori J., Baselga J., Villanueva J. (2013). Unconventional secretion is a major contributor of cancer cell line secretomes. Mol. Cell Proteomics.

[3] Zhang M., Liu L., Lin X., Wang Y., Li Y., Guo Q. (2020). A translocation pathway for vesicle-mediated unconventional protein secretion. Cell.

[4] Phulphagar K., Kuhn L.I., Ebner S., Frauenstein A., Swietlik J.J., Rieckmann J. (2021). Proteomics reveals distinct mechanisms regulating the release of cytokines and alarmins during pyroptosis. Cell Rep..

[5] Meissner F., Scheltema R.A., Mollenkopf H.J., Mann M. (2013). Direct proteomic quantification of the secretome of activated immune cells. Science.

[6] Dong L.F., Kovarova J., Bajzikova M., Bezawork-Geleta A., Svec D., Endaya B. (2017). Horizontal transfer of whole mitochondria restores tumorigenic potential in mitochondrial DNA-deficient cancer cells. Elife.

[7] Hurwitz S.N., Rider M.A., Bundy J.L., Liu X., Singh R.K., Meckes D.G. (2016). Proteomic profiling of NCI-60 extracellular vesicles uncovers common protein cargo and cancer type-specific biomarkers. Oncotarget.

[8] Islam M.N., Das S.R., Emin M.T., Wei M., Sun L., Westphalen K. (2012). Mitochondrial transfer from bone-marrow-derived stromal cells to pulmonary alveoli protects against acute lung injury. Nat. Med..

[9] Spees J.L., Olson S.D., Whitney M.J., Prockop D.J. (2006). Mitochondrial transfer between cells can rescue aerobic respiration. Proc. Natl. Acad. Sci. U. S. A..

[10] Todkar K., Chikhi L., Desjardins V., El-Mortada F., Pepin G., Germain M. (2021). Selective packaging of mitochondrial proteins into extracellular vesicles prevents the release of mitochondrial DAMPs. Nat. Commun..

[11] Steringer J.P., Muller H.M., Nickel W. (2015). Unconventional secretion of fibroblast growth factor 2--a novel type of protein translocation across membranes?. J. Mol. Biol..

[12] Planque C., Kulasingam V., Smith C.R., Reckamp K., Goodglick L., Diamandis E.P. (2009). Identification of five candidate lung cancer biomarkers by proteomics analysis of conditioned media of four lung cancer cell lines. Mol. Cell Proteomics.

[13] Makridakis M., Vlahou A. (2010). Secretome proteomics for discovery of cancer biomarkers. J. Proteomics.

[14] Ranganath S.H., Levy O., Inamdar M.S., Karp J.M. (2012). Harnessing the mesenchymal stem cell secretome for the treatment of cardiovascular disease. Cell Stem Cell.

[15] Carvalho M.M., Teixeira F.G., Reis R.L., Sousa N., Salgado A.J. (2011). Mesenchymal stem cells in the umbilical cord: phenotypic characterization, secretome and applications in central nervous system regenerative medicine. Curr. Stem Cell Res. Ther..

[16] Kim K., Kim K.H. (2020). Targeting of secretory proteins as a therapeutic strategy for treatment of Nonalcoholic Steatohepatitis (NASH). Int. J. Mol. Sci..

[17] Pardo M., Roca-Rivada A., Seoane L.M., Casanueva F.F. (2012). Obesidomics: contribution of adipose tissue secretome analysis to obesity research. Endocrine.

[18] Knecht S., Eberl H.C., Bantscheff M. (2022). Interval-based secretomics unravels acute-phase response in hepatocyte model systems. Mol. Cell Proteomics.

[19] Bantscheff M., Lemeer S., Savitski M.M., Kuster B. (2012). Quantitative mass spectrometry in proteomics: critical review update from 2007 to the present. Anal. Bioanal. Chem..

[20] Rozanova S., Barkovits K., Nikolov M., Schmidt C., Urlaub H., Marcus K. (2021). Quantitative mass spectrometry-based proteomics: an overview. Methods Mol. Biol..

[21] Bruderer R., Bernhardt O.M., Gandhi T., Miladinovic S.M., Cheng L.Y., Messner S. (2015). Extending the limits of quantitative proteome profiling with data-independent acquisition and application to acetaminophen-treated three-dimensional liver microtissues. Mol. Cell Proteomics.

[22] Gillet L.C., Navarro P., Tate S., Rost H., Selevsek N., Reiter L. (2012). Targeted data extraction of the MS/MS spectra generated by data-independent acquisition: a new concept for consistent and accurate proteome analysis. Mol. Cell Proteomics.

[23] Ludwig C., Gillet L., Rosenberger G., Amon S., Collins B.C., Aebersold R. (2018). Data-independent acquisition-based SWATH-MS for quantitative proteomics: a tutorial. Mol. Syst. Biol..

[24] Tushaus J., Muller S.A., Kataka E.S., Zaucha J., Sebastian Monasor L., Su M. (2020). An optimized quantitative proteomics method establishes the cell type-resolved mouse brain secretome. EMBO J..

[25] Deshmukh A.S., Cox J., Jensen L.J., Meissner F., Mann M. (2015). Secretome analysis of lipid-induced insulin resistance in skeletal muscle cells by a combined experimental and bioinformatics workflow. J. Proteome Res..

[26] Mendez O., Villanueva J. (2015). Challenges and opportunities for cell line secretomes in cancer proteomics. Proteomics Clin. Appl..

[27] Mbeunkui F., Fodstad O., Pannell L.K. (2006). Secretory protein enrichment and analysis: an optimized approach applied on cancer cell lines using 2D LC-MS/MS. J. Proteome Res..

[28] Brown K.J., Formolo C.A., Seol H., Marathi R.L., Duguez S., An E. (2012). Advances in the proteomic investigation of the cell secretome. Expert Rev. Proteomics.

[29] Hathout Y. (2007). Approaches to the study of the cell secretome. Expert Rev. Proteomics.

[30] Chevallet M., Diemer H., Van Dorssealer A., Villiers C., Rabilloud T. (2007). Toward a better analysis of secreted proteins: the example of the myeloid cells secretome. Proteomics.

[31] Mukherjee P., Mani S. (2013). Methodologies to decipher the cell secretome. Biochim. Biophys. Acta.

[32] Kleifeld O., Doucet A., auf dem Keller U., Prudova A., Schilling O., Kainthan R.K. (2010). Isotopic labeling of terminal amines in complex samples identifies protein N-termini and protease cleavage products. Nat. Biotechnol..

[33] Ong S.E., Mann M. (2006). A practical recipe for stable isotope labeling by amino acids in cell culture (SILAC). Nat. Protoc..

[34] Kuhn P.H., Koroniak K., Hogl S., Colombo A., Zeitschel U., Willem M. (2012). Secretome protein enrichment identifies physiological BACE1 protease substrates in neurons. EMBO J..

[35] Faca V.M., Ventura A.P., Fitzgibbon M.P., Pereira-Faca S.R., Pitteri S.J., Green A.E. (2008). Proteomic analysis of ovarian cancer cells reveals dynamic processes of protein secretion and shedding of extra-cellular domains. PLoS One.

[36] Imami K., Sugiyama N., Tomita M., Ishihama Y. (2010). Quantitative proteome and phosphoproteome analyses of cultured cells based on SILAC labeling without requirement of serum dialysis. Mol. Biosyst..

[37] Eichelbaum K., Winter M., Berriel Diaz M., Herzig S., Krijgsveld J. (2012). Selective enrichment of newly synthesized proteins for quantitative secretome analysis. Nat. Biotechnol..

[38] Pernemalm M., Lewensohn R., Lehtio J. (2009). Affinity prefractionation for MS-based plasma proteomics. Proteomics.

[39] Granger J., Siddiqui J., Copeland S., Remick D. (2005). Albumin depletion of human plasma also removes low abundance proteins including the cytokines. Proteomics.

[40] Weng Y., Sui Z., Shan Y., Jiang H., Zhou Y., Zhu X. (2016). In-depth proteomic quantification of cell secretome in serum-containing conditioned medium. Anal. Chem..

[41] Han D., Jin J., Woo J., Min H., Kim Y. (2014). Proteomic analysis of mouse astrocytes and their secretome by a combination of FASP and StageTip-based, high pH, reversed-phase fractionation. Proteomics.

[42] Poschmann G., Prescher N., Stuhler K. (2021). Quantitative MS workflow for a high-quality secretome analysis by a quantitative secretome-proteome comparison. Methods Mol. Biol..

[43] Xie L., Tsaprailis G., Chen Q.M. (2005). Proteomic identification of insulin-like growth factor-binding protein-6 induced by sublethal H2O2 stress from human diploid fibroblasts. Mol. Cell Proteomics.

[44] Rodrigues J.G., Balmana M., Macedo J.A., Pocas J., Fernandes A., de-Freitas-Junior J.C.M. (2018). Glycosylation in cancer: selected roles in tumour progression, immune modulation and metastasis. Cell Immunol..

[45] Loke I., Kolarich D., Packer N.H., Thaysen-Andersen M. (2016). Emerging roles of protein mannosylation in inflammation and infection. Mol. Aspects Med..

[46] Tilvawala R., Nguyen S.H., Maurais A.J., Nemmara V.V., Nagar M., Salinger A.J. (2018). The rheumatoid arthritis-associated citrullinome. Cell Chem Biol..

[47] Romero V., Fert-Bober J., Nigrovic P.A., Darrah E., Haque U.J., Lee D.M. (2013). Immune-mediated pore-forming pathways induce cellular hypercitrullination and generate citrullinated autoantigens in rheumatoid arthritis. Sci. Transl. Med..

[48] Paprocka J., Jezela-Stanek A., Tylki-Szymanska A., Grunewald S. (2021). Congenital disorders of glycosylation from a neurological perspective. Brain Sci..

[49] Reily C., Stewart T.J., Renfrow M.B., Novak J. (2019). Glycosylation in health and disease. Nat. Rev. Nephrol..

[50] Schjoldager K.T., Narimatsu Y., Joshi H.J., Clausen H. (2020). Global view of human protein glycosylation pathways and functions. Nat. Rev. Mol. Cell Biol..

[51] Kristic J., Lauc G. (2017). Ubiquitous importance of protein glycosylation. Methods Mol. Biol..

[52] Drake P.M., Cho W., Li B., Prakobphol A., Johansen E., Anderson N.L. (2010). Sweetening the pot: adding glycosylation to the biomarker discovery equation. Clin. Chem..

[53] Ohtsubo K., Marth J.D. (2006). Glycosylation in cellular mechanisms of health and disease. Cell.

[54] Boersema P.J., Geiger T., Wisniewski J.R., Mann M. (2013). Quantification of the N-glycosylated secretome by super-SILAC during breast cancer progression and in human blood samples. Mol. Cell Proteomics.

[55] Zhao J., Qiu W., Simeone D.M., Lubman D.M. (2007). N-linked glycosylation profiling of pancreatic cancer serum using capillary liquid phase separation coupled with mass spectrometric analysis. J. Proteome Res..

[56] Liu T., Qian W.J., Gritsenko M.A., Camp D.G., Monroe M.E., Moore R.J. (2005). Human plasma N-glycoproteome analysis by immunoaffinity subtraction, hydrazide chemistry, and mass spectrometry. J. Proteome Res..

[57] Zhang H., Li X.J., Martin D.B., Aebersold R. (2003). Identification and quantification of N-linked glycoproteins using hydrazide chemistry, stable isotope labeling and mass spectrometry. Nat. Biotechnol..

[58] Frost D.C., Li L. (2014). Recent advances in mass spectrometry-based glycoproteomics. Adv. Protein Chem. Struct. Biol..

[59] Alley W.R., Mann B.F., Novotny M.V. (2013). High-sensitivity analytical approaches for the structural characterization of glycoproteins. Chem. Rev..

[60] Riley N.M., Bertozzi C.R., Pitteri S.J. (2021). A pragmatic guide to enrichment strategies for mass spectrometry-based glycoproteomics. Mol. Cell Proteomics.

[61] Jiao J., Zhang H., Reinhold V.N. (2011). High performance IT-MS sequencing of glycans (Spatial Resolution of Ovalbumin Isomers). Int. J. Mass Spectrom..

[62] Bowman M.J., Zaia J. (2010). Comparative glycomics using a tetraplex stable-isotope coded tag. Anal. Chem..

[63] Walker S.H., Budhathoki-Uprety J., Novak B.M., Muddiman D.C. (2011). Stable-isotope labeled hydrophobic hydrazide reagents for the relative quantification of N-linked glycans by electrospray ionization mass spectrometry. Anal. Chem..

[64] Klement E., Medzihradszky K.F. (2017). Extracellular protein phosphorylation, the neglected side of the modification. Mol. Cell Proteomics.

[65] Yalak G., Vogel V. (2012). Extracellular phosphorylation and phosphorylated proteins: not just curiosities but physiologically important. Sci. Signal..

[66] Tagliabracci V.S., Wiley S.E., Guo X., Kinch L.N., Durrant E., Wen J. (2015). A single kinase generates the majority of the secreted phosphoproteome. Cell.

[67] Simpson M.A., Hsu R., Keir L.S., Hao J., Sivapalan G., Ernst L.M. (2007). Mutations in FAM20C are associated with lethal osteosclerotic bone dysplasia (Raine syndrome), highlighting a crucial molecule in bone development. Am. J. Hum. Genet..

[68] Lichtenthaler S.F., Lemberg M.K., Fluhrer R. (2018). Proteolytic ectodomain shedding of membrane proteins in mammals-hardware, concepts, and recent developments. EMBO J..

[69] Parks W.C., Wilson C.L., Lopez-Boado Y.S. (2004). Matrix metalloproteinases as modulators of inflammation and innate immunity. Nat. Rev. Immunol..

[70] Hu J., Van den Steen P.E., Sang Q.X., Opdenakker G. (2007). Matrix metalloproteinase inhibitors as therapy for inflammatory and vascular diseases. Nat. Rev. Drug Discov..

[71] Ishikawa H.O., Xu A., Ogura E., Manning G., Irvine K.D. (2012). The Raine syndrome protein FAM20C is a Golgi kinase that phosphorylates bio-mineralization proteins. PLoS One.

[72] Harada H., Farhani N., Wang X.F., Sugita S., Charish J., Attisano L. (2019). Extracellular phosphorylation drives the formation of neuronal circuitry. Nat. Chem. Biol..

[73] Urban J. (2022). A review on recent trends in the phosphoproteomics workflow. From sample preparation to data analysis. Anal. Chim. Acta.

[74] Lee C.Y., Wang D., Wilhelm M., Zolg D.P., Schmidt T., Schnatbaum K. (2018). Mining the human tissue proteome for protein citrullination. Mol Cell Proteomics.

[75] Lewallen D.M., Bicker K.L., Subramanian V., Clancy K.W., Slade D.J., Martell J. (2015). Chemical proteomic platform to identify citrullinated proteins. ACS Chem. Biol..

[76] Zawadzka A.M., Schilling B., Cusack M.P., Sahu A.K., Drake P., Fisher S.J. (2014). Phosphoprotein secretome of tumor cells as a source of candidates for breast cancer biomarkers in plasma. Mol. Cell Proteomics.

[77] Scilabra S.D., Pigoni M., Pravata V., Schatzl T., Muller S.A., Troeberg L. (2018). Increased TIMP-3 expression alters the cellular secretome through dual inhibition of the metalloprotease ADAM10 and ligand-binding of the LRP-1 receptor. Sci. Rep..

[78] Yang C.Y., Troeberg L., Scilabra S.D. (2020). Quantitative mass spectrometry-based secretome analysis as a tool to investigate metalloprotease and TIMP activity. Methods Mol. Biol..

[79] Hemming M.L., Elias J.E., Gygi S.P., Selkoe D.J. (2009). Identification of beta-secretase (BACE1) substrates using quantitative proteomics. PLoS One.

[80] Blobel G., Dobberstein B. (1975). Transfer of proteins across membranes. I. Presence of proteolytically processed and unprocessed nascent immunoglobulin light chains on membrane-bound ribosomes of murine myeloma. J. Cell Biol..

[81] Teufel F., Almagro Armenteros J.J., Johansen A.R., Gislason M.H., Pihl S.I., Tsirigos K.D. (2022). SignalP 6.0 predicts all five types of signal peptides using protein language models. Nat. Biotechnol..

[82] Dimou E., Nickel W. (2018). Unconventional mechanisms of eukaryotic protein secretion. Curr. Biol..

[83] Cohen M.J., Chirico W.J., Lipke P.N. (2020). Through the back door: unconventional protein secretion. Cell Surf..

[84] Alves P., Arnold R.J., Clemmer D.E., Li Y., Reilly J.P., Sheng Q. (2008). Fast and accurate identification of semi-tryptic peptides in shotgun proteomics. Bioinformatics.

[85] Gessulat S., Schmidt T., Zolg D.P., Samaras P., Schnatbaum K., Zerweck J. (2019). Prosit: proteome-wide prediction of peptide tandem mass spectra by deep learning. Nat Methods.

[86] Griswold A.R., Cifani P., Rao S.D., Axelrod A.J., Miele M.M., Hendrickson R.C. (2019). A chemical strategy for protease substrate profiling. Cell Chem Biol..

[87] Weng S.S.H., Demir F., Ergin E.K., Dirnberger S., Uzozie A., Tuscher D. (2019). Sensitive determination of proteolytic proteoforms in limited microscale proteome samples. Mol. Cell Proteomics.

[88] Waldera Lupa D.M., Kalfalah F., Safferling K., Boukamp P., Poschmann G., Volpi E. (2015). Characterization of skin aging-associated secreted proteins (SAASP) produced by dermal fibroblasts isolated from intrinsically aged human skin. J. Invest. Dermatol..

[89] Chen R., Kang R., Tang D. (2022). The mechanism of HMGB1 secretion and release. Exp. Mol. Med..

[90] de Seny D., Bianchi E., Baiwir D., Cobraiville G., Collin C., Deliege M. (2020). Proteins involved in the endoplasmic reticulum stress are modulated in synovitis of osteoarthritis, chronic pyrophosphate arthropathy and rheumatoid arthritis, and correlate with the histological inflammatory score. Sci. Rep..

[91] Rahmati M., Moosavi M.A., McDermott M.F. (2018). ER stress: a therapeutic target in rheumatoid arthritis?. Trends Pharmacol. Sci..

[92] Shields A.M., Thompson S.J., Panayi G.S., Corrigall V.M. (2012). Pro-resolution immunological networks: binding immunoglobulin protein and other resolution-associated molecular patterns. Rheumatology (Oxford).

[93] Grube L., Dellen R., Kruse F., Schwender H., Stuhler K., Poschmann G. (2018). Mining the secretome of C2C12 muscle cells: data dependent experimental approach to analyze protein secretion using label-free quantification and peptide based analysis. J. Proteome Res..

[94] Luo X., Liu Y., Wang R., Hu H., Zeng R., Chen H. (2011). A high-quality secretome of A549 cells aided the discovery of C4b-binding protein as a novel serum biomarker for non-small cell lung cancer. J. Proteomics.

[95] Stiess M., Wegehingel S., Nguyen C., Nickel W., Bradke F., Cambridge S.B. (2015). A dual SILAC proteomic labeling strategy for quantifying constitutive and cell-cell induced protein secretion. J. Proteome Res..

[96] Loei H., Tan H.T., Lim T.K., Lim K.H., So J.B., Yeoh K.G. (2012). Mining the gastric cancer secretome: identification of GRN as a potential diagnostic marker for early gastric cancer. J. Proteome Res..

[97] van Niel G., D'Angelo G., Raposo G. (2018). Shedding light on the cell biology of extracellular vesicles. Nat. Rev. Mol. Cell Biol..

[98] Johnstone R.M., Adam M., Hammond J.R., Orr L., Turbide C. (1987). Vesicle formation during reticulocyte maturation. Association of plasma membrane activities with released vesicles (exosomes). J. Biol. Chem..

[99] Zitvogel L., Regnault A., Lozier A., Wolfers J., Flament C., Tenza D. (1998). Eradication of established murine tumors using a novel cell-free vaccine: dendritic cell-derived exosomes. Nat. Med..

[100] Raposo G., Nijman H.W., Stoorvogel W., Liejendekker R., Harding C.V., Melief C.J. (1996). B lymphocytes secrete antigen-presenting vesicles. J. Exp. Med..

[101] Mallegol J., Van Niel G., Lebreton C., Lepelletier Y., Candalh C., Dugave C. (2007). T84-intestinal epithelial exosomes bear MHC class II/peptide complexes potentiating antigen presentation by dendritic cells. Gastroenterology.

[102] Doyle L.M., Wang M.Z. (2019). Overview of extracellular vesicles, their origin, composition, purpose, and methods for exosome isolation and analysis. Cells.

[103] Keerthikumar S., Chisanga D., Ariyaratne D., Al Saffar H., Anand S., Zhao K. (2016). ExoCarta: a web-based compendium of exosomal cargo. J. Mol. Biol..

[104] Pathan M., Fonseka P., Chitti S.V., Kang T., Sanwlani R., Van Deun J. (2019). Vesiclepedia 2019: a compendium of RNA, proteins, lipids and metabolites in extracellular vesicles. Nucleic Acids Res..

[105] Rieckmann J.C., Geiger R., Hornburg D., Wolf T., Kveler K., Jarrossay D. (2017). Social network architecture of human immune cells unveiled by quantitative proteomics. Nat. Immunol..

[106] Frauenstein A., Meissner F. (2018). Quantitative proteomics of secreted proteins. Methods Mol. Biol..

[107] Schmudlach A., Felton J., Cipolla C., Sun L., Kennedy R.T., Dovichi N.J. (2016). Sample preparation protocol for bottom-up proteomic analysis of the secretome of the islets of Langerhans. Analyst.

[108] Pirkmajer S., Chibalin A.V. (2011). Serum starvation: caveat emptor. Am. J. Physiol. Cell Physiol..

[109] Cooper S. (2003). Reappraisal of serum starvation, the restriction point, G0, and G1 phase arrest points. FASEB J..

[110] Hasan N.M., Adams G.E., Joiner M.C. (1999). Effect of serum starvation on expression and phosphorylation of PKC-alpha and p53 in V79 cells: implications for cell death. Int. J. Cancer.

[111] Franko A., Hartwig S., Kotzka J., Ruoss M., Nussler A.K., Konigsrainer A. (2019). Identification of the secreted proteins originated from primary human hepatocytes and HepG2 cells. Nutrients.

[112] Ong S.E., Blagoev B., Kratchmarova I., Kristensen D.B., Steen H., Pandey A. (2002). Stable isotope labeling by amino acids in cell culture, SILAC, as a simple and accurate approach to expression proteomics. Mol. Cell Proteomics.

[113] Dieterich D.C., Link A.J., Graumann J., Tirrell D.A., Schuman E.M. (2006). Selective identification of newly synthesized proteins in mammalian cells using bioorthogonal noncanonical amino acid tagging (BONCAT). Proc. Natl. Acad. Sci. U. S. A..

[114] Muller S.A., Scilabra S.D., Lichtenthaler S.F. (2016). Proteomic substrate identification for membrane proteases in the brain. Front. Mol. Neurosci..

[115] Vargas-Diaz D., Altelaar M. (2022). Automated high-throughput method for the fast, robust, and reproducible enrichment of newly synthesized proteins. J. Proteome Res..

[116] Jewett J.C., Bertozzi C.R. (2010). Cu-free click cycloaddition reactions in chemical biology. Chem. Soc. Rev..

[117] Sletten E.M., Bertozzi C.R. (2011). From mechanism to mouse: a tale of two bioorthogonal reactions. Acc. Chem. Res..

[118] Schira-Heinen J., Grube L., Waldera-Lupa D.M., Baberg F., Langini M., Etemad-Parishanzadeh O. (2019). Pitfalls and opportunities in the characterization of unconventionally secreted proteins by secretome analysis. Biochim. Biophys. Acta Proteins Proteom..

[119] Kiick K.L., Saxon E., Tirrell D.A., Bertozzi C.R. (2002). Incorporation of azides into recombinant proteins for chemoselective modification by the Staudinger ligation. Proc. Natl. Acad. Sci. U. S. A..

[120] Kirschner F., Arnold-Schild D., Leps C., Łącki M.K., Klein M., Ludt A. (2023). Modulation of cellular transcriptome and proteome composition by azidohomoalanine – implications on click chemistry based secretome analysis. J. Mol. Med. (Berl).

[121] Serdaroglu A., Muller S.A., Schepers U., Brase S., Weichert W., Lichtenthaler S.F. (2017). An optimised version of the secretome protein enrichment with click sugars (SPECS) method leads to enhanced coverage of the secretome. Proteomics.

[122] Kim K.E., Park I., Kim J., Kang M.G., Choi W.G., Shin H. (2021). Dynamic tracking and identification of tissue-specific secretory proteins in the circulation of live mice. Nat. Commun..

[123] Roux K.J., Kim D.I., Raida M., Burke B. (2012). A promiscuous biotin ligase fusion protein identifies proximal and interacting proteins in mammalian cells. J. Cell Biol..

[124] Liu J., Jang J.Y., Pirooznia M., Liu S., Finkel T. (2021). The secretome mouse provides a genetic platform to delineate tissue-specific *in vivo* secretion. Proc. Natl. Acad. Sci. U. S. A..

[125] Wei W., Riley N.M., Yang A.C., Kim J.T., Terrell S.M., Li V.L. (2021). Cell type-selective secretome profiling *in vivo*. Nat. Chem. Biol..

[126] Droujinine I.A., Meyer A.S., Wang D., Udeshi N.D., Hu Y., Rocco D. (2021). Proteomics of protein trafficking by *in vivo* tissue-specific labeling. Nat. Commun..

[127] Lam S.S., Martell J.D., Kamer K.J., Deerinck T.J., Ellisman M.H., Mootha V.K. (2015). Directed evolution of APEX2 for electron microscopy and proximity labeling. Nat. Methods.

[128] Kotani N., Gu J., Isaji T., Udaka K., Taniguchi N., Honke K. (2008). Biochemical visualization of cell surface molecular clustering in living cells. Proc. Natl. Acad. Sci. U. S. A..

[129] Branon T.C., Bosch J.A., Sanchez A.D., Udeshi N.D., Svinkina T., Carr S.A. (2018). Efficient proximity labeling in living cells and organisms with TurboID. Nat. Biotechnol..

[130] Cronan J.E. (2005). Targeted and proximity-dependent promiscuous protein biotinylation by a mutant Escherichia coli biotin protein ligase. J. Nutr. Biochem..

[131] Tanzer M.C., Frauenstein A., Stafford C.A., Phulphagar K., Mann M., Meissner F. (2020). Quantitative and dynamic catalogs of proteins released during apoptotic and necroptotic cell death. Cell Rep..

[132] Deshmukh A.S., Peijs L., Beaudry J.L., Jespersen N.Z., Nielsen C.H., Ma T. (2019). Proteomics-based comparative mapping of the secretomes of human Brown and white adipocytes reveals EPDR1 as a novel batokine. Cell Metab..

[133] Wang X.D., Hu R., Ding Q., Savage T.K., Huffman K.E., Williams N. (2019). Subtype-specific secretomic characterization of pulmonary neuroendocrine tumor cells. Nat. Commun..

[134] Ali Khan A., Hansson J., Weber P., Foehr S., Krijgsveld J., Herzig S. (2018). Comparative secretome analyses of primary murine white and Brown adipocytes reveal novel adipokines. Mol. Cell Proteomics.

[135] Eichelbaum K., Krijgsveld J. (2014). Rapid temporal dynamics of transcription, protein synthesis, and secretion during macrophage activation. Mol. Cell Proteomics.

[136] Shin J., Rhim J., Kwon Y., Choi S.Y., Shin S., Ha C.W. (2019). Comparative analysis of differentially secreted proteins in serum-free and serum-containing media by using BONCAT and pulsed SILAC. Sci. Rep..

[137] Witzke K.E., Rosowski K., Muller C., Ahrens M., Eisenacher M., Megger D.A. (2017). Quantitative secretome analysis of activated Jurkat cells using click chemistry-based enrichment of secreted glycoproteins. J. Proteome Res..

[138] Eichelbaum K., Krijgsveld J. (2014). Combining pulsed SILAC labeling and click-chemistry for quantitative secretome analysis. Methods Mol. Biol..

[139] Chang Z., Duan Q., Yu C., Li D., Jiang H., Ge F. (2022). Proteomics and biochemical analyses of secreted proteins revealed a novel mechanism by which ADAM12S regulates the migration of gastric cancer cells. J. Proteome Res..

